# Chromosomal Aneuploidy Associated With Clinical Characteristics of Pregnancy Loss

**DOI:** 10.3389/fgene.2021.667697

**Published:** 2021-04-15

**Authors:** Chongjuan Gu, Kuanrong Li, Ru Li, Ling Li, Xiaojun Li, Xinyu Dai, Yaojuan He

**Affiliations:** ^1^Department of Obstetrics and Gynecology, Guangzhou Women and Children’s Medical Center, Guangzhou Medical University, Guangzhou, China; ^2^Institute of Pediatrics, Guangzhou Women and Children’s Medical Center, Guangzhou Medical University, Guangzhou, China; ^3^Prenatal Diagnostic Center, Guangzhou Women and Children’s Medical Center, Guangzhou Medical University, Guangzhou, China; ^4^Clinical Data Center, Guangzhou Women and Children’s Medical Center, Guangzhou Medical University, Guangzhou, China; ^5^School of Life Sciences, South China Normal University, Guangzhou, China

**Keywords:** chromosomal aneuploidy, products of conception, pregnancy loss, chromosomal microarray analysis, clinical characteristics

## Abstract

**Objective:**

Embryonic aneuploidy is found in about half of sporadic pregnancy losses and the associations between the chromosomal aneuploidy and clinical characteristics of pregnancy loss remain unclear. The aims of this study were to evaluate the associations between chromosomal aneuploidy of products of conception (POC) and clinical features of pregnancy loss.

**Methods:**

We conducted a retrospective cohort study including 1,102 women experienced singleton pregnancy loss and underwent chromosomal microarray analysis (CMA) detection of POC in our hospital. The results of molecular karyotypes and clinical features including maternal age, history of pregnancy loss, gestational age, vaginal bleeding and ultrasonographic findings were extracted from the medical records. χ^2^ test was used to compare categorical data between groups.

**Results:**

631 (57.26%) POC specimens were detected to be chromosomal aneuploidy. Aneuploid rates were significantly higher in women >35 years (*P* < 0.001) and pregnancy loss <11 gestational weeks (*P* = 0.044), but the rates of sex chromosome abnormalities and triploid were significantly higher in women ≤35 years (*P* < 0.001, *P* = 0.002) and the rates of viable autosomal trisomy and sex chromosome abnormalities were significantly high in those women with pregnancy loss ≥11 weeks (P < 0.001, *P* < 0.001). Aneuploid rate was overall similar between the sporadic and the recurrent pregnancy loss (RPL) (*P* = 0.404), but the rate of sex chromosome abnormalities was higher in women with sporadic pregnancy loss (*P* = 0.03). Aneuploid rates were higher in subjects with yolk sac or embryo than in those without (*P* < 0.001 and *P* = 0.001).

**Conclusion:**

Advanced maternal age is mainly associated with autosomal trisomy, while sex chromosome abnormalities and triploid might be more likely to occur in younger women. Aneuploidy rates might be no association with previous pregnancy loss except for sex chromosome abnormalities. Pregnancy loss without yolk sac or embryo might be less related to embryonic aneuploidy, and other factors should be emphasized.

## Introduction

It is well known that 15–20% of clinically recognized pregnancies end in pregnancy loss, and approximately 1–2% of couples experience recurrent pregnancy loss (RPL) (Practice Committee of the American Society for Reproductive Medicine, 2012; The Eshre Guideline Group on RPL et al., 2018). Embryonic/fetal chromosomal abnormalities are found in about 50% of early sporadic pregnancy loss, and aneuploidy is the most frequently observed abnormality (Hassold et al., 1980; Sahoo et al., 2017). The chromosomal analysis of products of conception (POC) is not a routine practice for women who have pregnancy loss. However, accurate identification of the genetic characteristics of a pregnancy loss can provide important information for medical management, reproductive counseling, and supportive patient care (Menasha et al., 2005; Zhang et al., 2009).

Although G-banding karyotyping has been used for many years to evaluate samples of POC, the high rate of culture failure and maternal cell contamination are two primary limitations (Lomax et al., 2000; van den Berg et al., 2012). Evaluation of aneuploidy by fluorescence *in situ* hybridization (FISH) analysis avoids the above defects, but is significantly limited to a smaller number of targeted chromosomes (Shearer et al., 2011; Russo et al., 2016). Chromosomal microarray analysis (CMA) using genome-wide oligonucleotide or single-nucleotide polymorphism (SNP)-based arrays has replaced karyotyping in some prenatal diagnostic applications owing to its higher resolution and detection rates of chromosomal abnormalities (Hillman et al., 2011; Dhillon et al., 2014), but it can’t be widely used because of high cost and technical requirements.

The advanced maternal age is the most convincing clinical factor of pregnancy loss because of age-related meiotic errors in oogenesis (Grande et al., 2012; Hardy et al., 2016). However, it is unclear whether all the aneuploid karyotypes of POC are more frequent in women of advanced age. Another possible factor is maternal history of RPL. However, a decreased chromosomal aneuploid rate of POC in women with RPL has been reported in some but not all studies (Ogasawara et al., 2000; Morikawa et al., 2004; Sullivan et al., 2004; Goldstein et al., 2017). In addition, pregnancy loss with different chromosomal karyotypes may have disparate developmental potentials (Andrews et al., 1984; Minelli et al., 1993). Ultrasonographic findings of pregnancy loss show a range of development arrest stages: an empty sac with or without yolk sac, having little evidence of an embryo or a proper crown-rump length (CRL). Several studies have reported that the presence of a fetal pole or fetal cardiac activity related to the frequency of chromosomal abnormalities in pregnancy loss (Munoz et al., 2010; Cheng et al., 2014; Zhang et al., 2014; Romero et al., 2015). But two other studies had not found a relation between ultrasound findings and karyotypes (Bessho et al., 1995; Coulam et al., 1997).

Given the conflicting results of existing studies, we conducted a retrospective cohort study to investigate the associations between the molecular karyotypes of POC detected by CMA with clinical features, including the maternal age, history of pregnancy loss and the ultrasound findings.

## Materials and Methods

We conducted a retrospective, hospital-based cohort study at Guangzhou Women and Children’s Medical Center, a tertiary referral hospital in South China. The study protocol was approved by the ethics committee of the institute (2020-15001). In the cohort, women who were treated for pregnancy loss and underwent CMA test in our hospital between May 2016 and May 2020 were included. All patients provided a written informed consent for the tests and the inclusion of results in research. All women were clinically confirmed pregnancy by transvaginal ultrasound that detected an intrauterine gestational sac. Pregnancy loss was diagnosed by transvaginal ultrasound and blood β human chorionic gonadotropin according to the guideline (Doubilet et al., 2013). All patients underwent expectant management, or medical management (Mifepristone/Misoprostol) or dilation and curettage after the diagnosis of pregnancy loss. Fresh POC specimens were collected and villous tissue was carefully separated for the CMA detection. Maternal peripheral blood was obtained for the quantitative fluorescent-polymerase chain reaction (QF-PCR).

DNA was extracted using QIAamp DNA Blood and Tissue kits (Qiagen, Dusseldorf, Germany). All samples were tested for maternal cell contamination using QF-PCR based on short tandem repeat (STR) markers for chromosomes 13, 18, 21, and X, Y (Diego-Alvarez et al., 2005; Nagan et al., 2011). The CMA platform used Cyto Scan 750 K Array (Affymetrix Inc., Santa Clara, CA, United States) containing 550,000 oligonucleotide probes and 200,436 single nucleotides polymorphic (SNP) probes. Data were visualized and analyzed with the chromosome analysis Suite (ChAS) software (Affymetrix, Santa Clara, CA, United States) based on the GRCh37/hg19 assembly.

Demographic and baseline clinical data such as maternal age, history of pregnancy loss, gestational age of pregnancy loss and whether or not presenting vaginal bleeding was obtained from the clinical records. Ultrasonographic findings such as size of sac, with or without a yolk sac and CRL were also extracted from ultrasonic reports. Maternal age was classified into two groups: ≤35 and >35 years. With regard to history of previous pregnancy loss, the subjects were divided into two groups: sporadic (precious pregnancy loss < 2) and recurrent (precious pregnancy loss ≥2) pregnancy loss according to the guideline (The Eshre Guideline Group on RPL et al., 2018). According to gestational age while the embryo or fetus demising, the subjects were divided into two groups: <11 gestational weeks and ≥11 gestational weeks. The subjects were divided into two groups according to whether there was vaginal bleeding and whether yolk sac and embryo were found on ultrasound scan. On the basis of the embryonic/fetal size detected by ultrasound at pregnancy loss, the patients were divided into two groups: CRL ≤ 20 mm and >20 mm.

Clinical features were compared between the euploidy and aneuploidy groups by χ^2^ test or adjusted χ^2^ test. We further limited the analysis to subjects with aneuploidy to explore the associations that assumed to exist between specific aneuploidy karyotype with clinical features. *P* value < 0.05 was considered statistically significant. All statistical analyses were performed using Statistical Package for the Social Sciences for Windows, version 12.0 (SPSS, Inc., Chicago, IL, United States).

## Results

A total of 1,102 single pregnant women with pregnancy loss were included in the cohort. CMA test of fresh POC samples was successful in all cases, with 471 (42.74%) chromosomal euploid and 631 (57.26%) chromosomal aneuploid detected. The mean age of the euploid and aneuploid pregnancy loss women were 31.30 years (range 20–48 years) and 32.51 years (range 18–45 years) (*P* < 0.001), respectively. The mean gestational ages of the euploid and aneuploid groups were 11.1 weeks (range 4–34 weeks) and 9.7 weeks (range 4–28 weeks) (*P* < 0.001), respectively.

Table 1 illustrates euploid and aneuploid rates according to maternal age, gestational age, history of pregnancy loss, vaginal bleeding, and ultrasonographic features. The aneuploid rate was significantly higher in the women >35 years than in those ≤ 35 years (*P* < 0.001). There was no statistical difference in the rate of aneuploid between the sporadic and RPL (*P* = 0.404). The aneuploid rate of the group of pregnancy loss < 11 gestational weeks was statistically higher than those ≥11 gestational weeks (*P* = 0.044). The chromosomal aneuploid rate was not statistically different between the group presenting or not vaginal bleeding (*P* = 0.334). With respect to ultrasonographic features, the results showed that the rates of chromosomal aneuploid were significantly higher in subjects with yolk sac or embryo than in those without (*P* < 0.001, *P* = 0.001). In addition, the chromosomal aneuploid rate was significant higher in the group of CRL ≤ 20 mm than in the group of CRL >20 mm (*P* < 0.001).

**TABLE 1:** The comparison of clinical characteristics between euploid and aneuploid pregnancy loss.

		**Euploid *n* = 471 (42.74%)**	**Aneuploid *n* = 631 (57.25%)**	**P**
Maternal age (years)	≤35	391 (46.44)	451 (53.56)	<0.001
	>35	68 (27.76)	177 (72.24)	
Gestational age (week)	<11	287 (40.37)	424 (59.63)	0.044
	≥11	173 (46.76)	197 (53.24)	
History of pregnancy loss	Sporadic	397 (42.37)	540 (57.63)	0.404
	Recurrent	65 (46.1)	76 (53.9)	
Vaginal bleeding	Yes	153 (40.58)	224 (59.42)	0.334
	No	305 (43.63)	394 (56.37)	
Yolk sac	Yes	307 (39.51)	470 (60.49)	<0.001
	No	60 (64.52)	33 (35.48)	
Embryo	Yes	285 (37.95)	466 (62.05)	0.001
	No	72 (52.94)	64 (47.06)	
CRL (mm)	≤20	100 (32.05)	212 (67.95)	<0.001
	>20	42 (53.16)	37 (46.84)	

*CRL, crown-rump length.*

**FIGURE 1:**
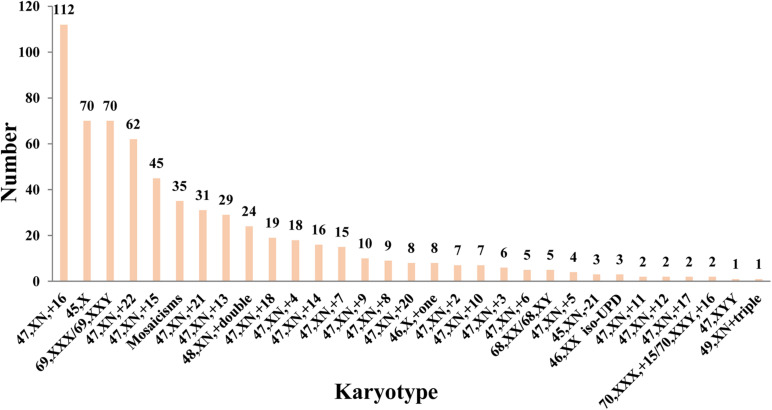
Chromosomal aneuploid spectrum of POC in 631 pregnancy loss.

Figure 1 displays aneuploid spectrum detected in the cohort, the most common aneuploid karyotype was trisomy 16 following with 45, X and triploid. Autosomal trisomy accounted for 64.82% (409/631) in the all POC aneuploidy. It is noteworthy that almost all chromosome trisomy or monosomy was found except for chromosomes 1 and 19.

We then conducted subgroup analysis to explore the associations between the aneuploid karyotype and the clinical features (Table 2). The frequency of viable autosomal trisomy was significantly increased in the women >35 years old (*P* = 0.019), gestational age ≥11 weeks (*P* < 0.001), and CRL >20 mm (*P* = 0.002), but was not associated with the history of pregnancy loss (*P* = 0.195). The rate of sex chromosome abnormalities was statistically increased in the group of maternal age ≤ 35 years old (*P* < 0.001), sporadic pregnancy loss (*P* = 0.003), gestational age ≥ 11 weeks (*P* < 0.001), and CRL > 20 mm (*P* < 0.001). The frequency of non-viable autosomal trisomy, accounted for 52.3% (330/631) of the all aneuploid karyotypes, was significantly high in the group of maternal age >35 years (*P* = 0.021), gestational age < 11 weeks (*P* < 0.001), and CRL ≤ 20 mm (*P* < 0.001), and was not associated with the history of pregnancy loss (*P* = 0.241). The frequency of two or more chromosomal abnormalities was increased in the group of <11 gestational weeks (*P* = 0.028), but not associated with maternal age (*P* = 0.062), history of pregnancy loss (*P* = 0.613), and CRL (*P* = 0.9). The rate of triploid was statistically higher in maternal age ≤ 35 years (*P* = 0.002), but was not associated with other clinical features. The frequency of mosaicisms showed no statistical difference between all those subgroups.

**TABLE 2:** The association between the frequency of chromosomal aneuploidy with clinical characteristics.

		**Maternal age**			**History**			**Gestational Age**			**CRL**		
**Karyotype**	**Number**	**≤35 Y**	**>35 Y**	**P**	**Sporadic**	**Recurrent**	**P**	**<11 W**	**≥11 W**	**p**	**≤20 mm**	**>20 mm**	**p**
Viable autosomal trisomy^a^	79	48 (10.64)	31 (17.51)	**0.019**	71 (13.15)	6 (7.89)	0.195	26 (6.13)	53 (26.9)	**<0.001**	19 (8.96)	10 (27.03)	**0.002**
Sex chromosome abnormalies^b^	71	64 (14.19)	7 (3.95)	**<0.001**	67 (12.41)	3 (3.95)	**0.03**	18 (4.25)	53 (26.9)	**<0.001**	17 (8.02)	16 (43.24)	**<0.001**
Non-viable autosomal trisomy^c^	330	224 (49.67)	106 (59.89)	**0.021**	281 (52.04)	45 (59.21)	0.241	266 (62.74)	58 (29.44)	**<0.001**	133 (62.74)	2 (5.41)	**<0.001**
Two or more chromosome anomalies^d^	33	19 (4.21)	14 (7.91)	0.062	28 (5.19)	5 (6.58)	0.613	28 (6.84)	5 (2.54)	**0.028**	5 (2.36)	1 (2.7)	0.9
Triploid	77	67 (14.86)	10 (5.65)	**0.002**	62 (11.48)	12 (15.79)	0.279	55 (12.97)	20 (10.15)	0.316	27 (12.74)	8 (21.62)	0.151
Mosaicisms	35	26 (5.76)	9 (5.08)	0.738	28 (5.19)	5 (6.58)	0.613	27 (6.37)	8 (4.06)	0.246	11 (5.19)	0 (0)	0.156

*CRL, crown-rump length.*

*^*a*^Trisomy 13, 18, or 21.*

*^*b*^45, X; 47, XXY.*

*^*c*^Autosomal trisomy excepting for Trisomy 13, 18, or 21.*

*^*d*^48, XN, +double and 49, XN, +triple.*

*The bold values mean statistically different results.*

## Discussion

An increased detection rate of chromosomal aneuploidy has been reported while using CMA to analyze the POC (Dhillon et al., 2014). In present study, aneuploid rate of the POC was 57.26% detected by CMA. In accordance with previous reports (Eiben et al., 1990; Ozawa et al., 2019), our results demonstrate that pregnancy loss in women over 35 years of age is associated with a higher chromosomal aneuploid rate. However, this characteristic only presents in autosomal trisomy, which accounted for 64.54% of the aneuploidy in our series, on the contrary, the triploid and the sex chromosome abnormalities, especially 45, X, are more likely to occur in women ≤ 35 years old, and the two or more chromosome abnormalities and mosaicisms might not be related to maternal age. It is well known that chromosome trisomy mainly results from un-separated chromosome in oogenesis, which is related to advanced maternal age^13,14^. Triploid is supposed to result from incorrect ploidy at fertilization, and it may be diandry (two paternal sets) or digyny (two maternal sets) (Marton et al., 1999). Monosomy X is thought more likely to be caused by meiotic error of the father rather than the mother (Hassold et al., 1988; Segawa et al., 2017). Further research including couples’ age should be conducted to confirm whether triploid and monosomy X are related to younger maternal age.

Overall, our results showed that chromosomal aneuploidy was more likely to occur in women with pregnancy loss ≤ 11 gestational weeks and CRL < 20 mm. However, this feature was just demonstrated in non-viable autosomal trisomy and two or more chromosome abnormalities, contributed 57.5% (363/631) of the aneuploid karyotypes in this cohort, which resulting in earlier embryo demise is reasonable. Meanwhile, in those viable aneuploid such as trisomy 13, 18, 21, monosomy X and 47, XXY, pregnancy loss occurs in later gestational age. And the triploid and mosaicisms showed no difference between different gestational ages of pregnancy loss.

Three previous studies reported no difference of the aneuploid rate between sporadic and RPL (Coulam et al., 1996;Stephenson et al., 2002; Grande et al., 2012), but Ogasawara et al. (2000) and Sullivan et al. (2004) described decreased rates of chromosomal abnormalities in RPL. From out data, in overall, the aneuploid rate is no difference between sporadic and RPL, but sex chromosomal abnormalities might occur less frequently in RPL.

With regard to the correlation between ultrasound embryonic pole and chromosomal karyotype, several previous studies have reported contradictory results (Lathi et al., 2007; Munoz et al., 2010; Cheng et al., 2014; Romero et al., 2015; Ouyang et al., 2016). The discrepancy may result from the difference in population, sample size, and laboratory method. Our study separately investigated the correlation between molecular karyotype with yolk sac and embryo in a larger cohort, and the results suggest that chromosomal aneuploidy is more likely to occur in pregnancy loss with yolk sac or embryo in ultrasound scan than in those without. Pregnancy loss is a complex condition caused by multi-etiological factors, and women with very early pregnancy loss, those without yolk sac or embryo in ultrasound scan, might need to consider more in other factors than chromosomal aneuploidy.

Another noteworthy result is that among all the 409 detected trisomy, chromosome 1 and 19 were not detected in trisomy or monosomy. Through literature review, we realize that trisomy 1 has only been reported in four pregnancy loss cases (Hanna et al., 1997; Dunn et al., 2001; Banzai et al., 2004; Vicic et al., 2008), and trisomy 9 has been reported in seven pregnancy loss cases (Hoshi et al., 1997; Choi et al., 2014; Hardy et al., 2016; Ouyang et al., 2016; Segawa et al., 2017). It is unclear that why trisomy 1 and trisomy 19 occur rarely, it may be related to the mechanism of gamete meiosis. Chromosome 1 is the biggest chromosome in human, which might not be prone to chromosome non-separation in the process of gamete meiosis. All those enigmas need more research in the future.

The strengths of this study include its population-based nature and relatively large sample size. The methods for both sample retrieving and CMA testing were uniform for all the study populations, in contrast to some series including cases in different laboratories. In addition, this is the first study to extensively investigate the associations between a wide spectrum of chromosomal aneuploids and clinical features of pregnancy loss. However, our study has two limitations. Firstly, we were unable to analyze the association between gender of POC and chromosomal euploidy since gender identification of embryo/fetus is not permitted by regulations in China. Secondly, we were unable to confirm the potential impact of *in vitro* fertilization (IVF) treatment on chromosomal euploidy as data on IVF were largely missing.

In conclusion, advanced maternal age mainly relates to increased autosomal trisomy, but sex chromosome abnormalities and triploid might more likely occur in younger women. The rate of chromosomal aneuploidy might not be different in history of previous pregnancy loss except for sex chromosome abnormalities, which might be less likely to occur in RPL. Pregnancy loss without yolk sac or embryo might be less relates to embryonic aneuploidy and other factors should be emphasized in the causes of the pregnancy loss. This study not only provides evidence for patient genetic counseling and management, but also provides hints for future research in exploring the mechanism of high aneuploidy rate in human embryo.

## Data Availability Statement

The raw data supporting the conclusions of this article will be made available by the authors, without undue reservation.

## Ethics Statement

The studies involving human participants were reviewed and approved by the Ethical Committee of the Guangzhou Women and Children’s Medical Center (2020-15001). The patients/participants provided their written informed consent to participate in this study.

## Author Contributions

CG and YH drafted the manuscript. CG and KL contributed to the design of the research. LL and XL contributed to the development of the statistical plan and statistical analysis of the data. CG contributed to the conception of the study. XD and RL contributed to data management and prepared the retrospective data for analysis. All authors reviewed, read and approved the final manuscript.

## Conflict of Interest

The authors declare that the research was conducted in the absence of any commercial or financial relationships that could be construed as a potential conflict of interest.
